# Rhesus TRIM5α Disrupts the HIV-1 Capsid at the Inter­Hexamer Interfaces

**DOI:** 10.1371/journal.ppat.1002009

**Published:** 2011-03-24

**Authors:** Gongpu Zhao, Danxia Ke, Thomas Vu, Jinwoo Ahn, Vaibhav B. Shah, Ruifeng Yang, Christopher Aiken, Lisa M. Charlton, Angela M. Gronenborn, Peijun Zhang

**Affiliations:** 1 Department of Structural Biology, University of Pittsburgh School of Medicine, Pittsburgh, Pennsylvania, United States of America; 2 Department of Microbiology and Immunology, Vanderbilt University School of Medicine, Nashville, Tennessee, United States of America; University of Geneva, Switzerland

## Abstract

TRIM proteins play important roles in the innate immune defense against retroviral infection, including human immunodeficiency virus type-1 (HIV-1). Rhesus macaque TRIM5α (TRIM5α_rh_) targets the HIV-1 capsid and blocks infection at an early post-entry stage, prior to reverse transcription. Studies have shown that binding of TRIM5α to the assembled capsid is essential for restriction and requires the coiled-coil and B30.2/SPRY domains, but the molecular mechanism of restriction is not fully understood. In this study, we investigated, by cryoEM combined with mutagenesis and chemical cross-linking, the direct interactions between HIV-1 capsid protein (CA) assemblies and purified TRIM5α_rh_ containing coiled-coil and SPRY domains (CC-SPRY_rh_). Concentration-dependent binding of CC-SPRY_rh_ to CA assemblies was observed, while under equivalent conditions the human protein did not bind. Importantly, CC-SPRY_rh_, but not its human counterpart, disrupted CA tubes in a non-random fashion, releasing fragments of protofilaments consisting of CA hexamers without dissociation into monomers. Furthermore, such structural destruction was prevented by inter-hexamer crosslinking using P207C/T216C mutant CA with disulfide bonds at the CTD-CTD trimer interface of capsid assemblies, but not by intra-hexamer crosslinking via A14C/E45C at the NTD-NTD interface. The same disruption effect by TRIM5α_rh_ on the inter-hexamer interfaces also occurred with purified intact HIV-1 cores. These results provide insights concerning how TRIM5α disrupts the virion core and demonstrate that structural damage of the viral capsid by TRIM5α is likely one of the important components of the mechanism of TRIM5α-mediated HIV-1 restriction.

## Author Summary

The cellular protein TRIM5α is a host cell restriction factor that blocks HIV-1 infection in Rhesus macaque cells by targeting the viral capsid. Here, we show that direct binding of a TRIM5α protein, consisting of the coiled-coil and B30.2/SPRY domains, to the viral capsid results in disruption of the surface lattice and fragmentation of the capsid, specifically at inter-hexamer interfaces. Our results reinforce the notion that structural damage of the viral capsid by TRIM5α is central to the mechanism of TRIM5α-mediated HIV-1 restriction.

## Introduction

TRIM5α is an important component of the innate immune defense against retroviral infection, including human immunodeficiency virus type -1 (HIV-1) [1], [2], and numerous studies suggest that TRIM5α interacts with assembled capsids and induces premature capsid disassembly (uncoating), before reverse transcription takes place [3]–[6]. TRIM5α is a 56 kD protein comprising a tripartite motif (TRIM; with RING, B-box 2, and coiled-coil (CC) domains) followed by a C-terminal B30.2/SPRY domain [7]–[9]. Each of these domains plays distinct roles in the antiviral function of TRIM5α. The B30.2/SPRY domain binds to the viral capsid and determines the specificity of restriction, with sequence variation within this domain greatly impacting binding specificity [6], [10]–[16]. For example, a single amino acid change in human TRIM5α (TRIM5α_hu_), R332P, renders the protein capable of binding the HIV-1 capsid, causing it to behave like rhesus TRIM5α (TRIM5α_rh_) with regard to HIV-1 restriction [11], [17]. The CC domain is necessary and sufficient for TRIM5α homo-dimerization, and this is important for capsid binding and restriction [12], [18]–[20]. *In vitro*, specific recognition and binding to a hexagonal CA lattice requires both the CC and SPRY domains [19]. The B-box 2 domain is thought to be involved in higher-order structure formation and self-association, and its presence in the protein enhances TRIM5α binding to the capsid, compared to the CC-SPRY domains alone [21], [22]. Several mutations in the B-box 2 domain abrogate HIV-1 restriction by TRIM5α_rh_ [22]–[24]. The N-terminal RING domain is the least explored domain of TRIM5α. In general, RING domains are components of a particular class of E3 ubiquitin ligases that are involved in proteasome-mediated protein degradation (reviewed in [25]). TRIM5α exhibits E3 activity, but the role of the ubiquitin ligase activity in retrovirus restriction is unclear. Deletion of the N-terminal RING domain reduces, but does not abolish antiviral restriction [23], [26], and treatment of cells with proteasome inhibitors does not prevent restriction by TRIM5α [27]. However, proteasome activity is necessary for the TRIM5α-mediated block to reverse transcription [27], and engagement of restriction-sensitive virus cores results in proteasome-dependent degradation of TRIM5α [28]. Together, these data suggest that TRIM5α action in host restriction of retroviruses involves all of its domains.

The negative influence of TRIM5α on viral reverse transcription is well established [1], [3], [4], [6], [29], [30], however, the detailed mechanism of restriction has not been elucidated. TRIM5α binds to assembled complexes composed of the CA-NC region of Pr55^gag^, but does not significantly interact with monomeric or soluble CA protein [31]. Furthermore, mutations in CA that decrease capsid stability appear to reduce TRIM5α binding in target cells, as HIV-1 particles with unstable cores are less effective at saturating TRIM5α-mediated restriction [5]. Finally, recent studies using a recombinant TRIM5α_rh_ chimera, containing the RING domain of TRIM21, demonstrated that the hybrid protein binds to CA-NC tubular assemblies and causes shortening of the tubes [32], [33], or self-assembles into higher-order structures, enhanced by binding to a preformed CA-NC hexagonal template [34].

Here, we employed cryoEM to investigate the direct interactions of tubular HIV-1 capsid assemblies and purified HIV-1 cores with the TRIM5α_rh_ CC-SPRY protein and the structural consequences of TRIM5α_rh_ CC-SPRY binding. We demonstrate that TRIM5α_rh_ binding disrupts the tubes and creates non-random fragments. Specific inter-hexamer interfaces are preferentially broken, resulting in strings of subunits that are held together by the CA-CTD dimer. We further demonstrate that disruption by TRIM5α_rh_ of purified HIV-1 cores also occurred preferentially at the inter-hexamer interfaces. Our data suggest that TRIM5α_rh_-mediated HIV-1 restriction involves direct engagement of the viral capsid, and structural damage to the capsid is likely one of the key components in this event.

## Results and Discussion

### Expression, purification, and biophysical characterization of recombinant TRIM5α CC-SPRY

**Figure 1:**
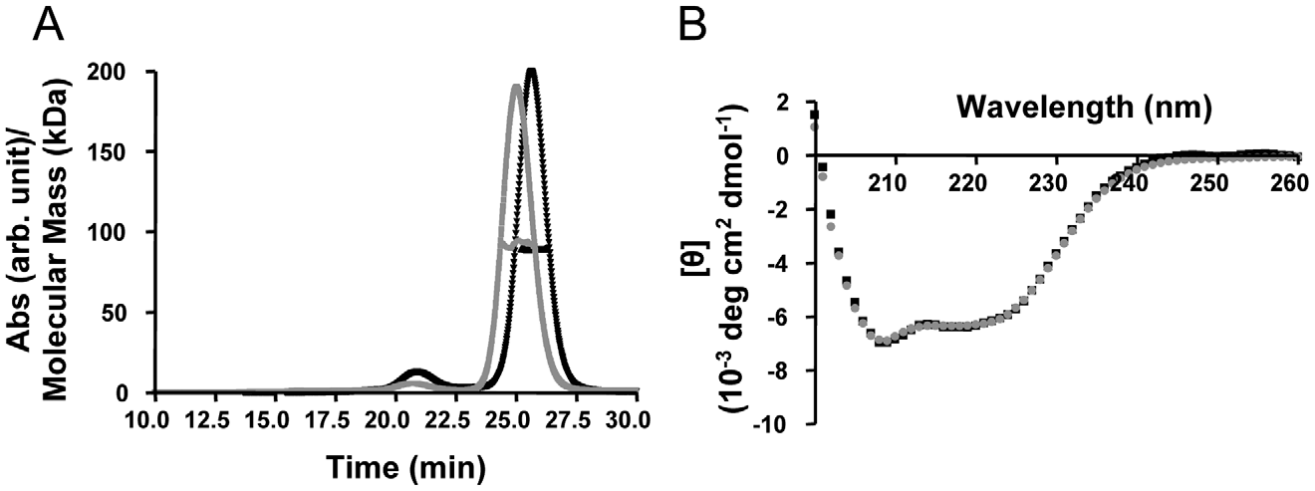
Biophysical characterization of recombinant TRIM5α CC-SPRY. (A) Multi angle light scattering data. Elution profiles (A_280_ values) for rhesus monkey and human TRIM5α CC-SPRY proteins are shown in black and gray, respectively, and the calculated molecular masses obtained from the light scattering are shown with black and gray symbols across the peaks. (B) Superposition of the CD spectra of TRIM5α_rh_ CC-SPRY (black) and TRIM5α_hu_ CC-SPRY (gray).

To investigate the direct interactions between TRIM5α_rh_ and the HIV-1 capsid, we generated purified recombinant proteins. Full length, wild-type TRIM5α_rh_ has been quite difficult to obtain in sufficient quantities for biophysical and structural studies [32], [35]. Therefore, we tested the expression and solubility of a number of different TRIM5α_rh_ constructs, including one that comprises the CC-SPRY portion, by performing transient expression in SF9 insect cells. TRIM5α_rh_ CC-SPRY (residues 134–497) and TRIM5α_hu_ CC-SPRY (residues of 132–493) exhibited sufficient protein levels and solubility and, therefore, were selected for production in SF21 insect cells, using recombinant baculoviruses. The quaternary state of the purified recombinant human and rhesus TRIM5α CC-SPRY proteins was assessed by size exclusion chromatography in conjunction with in-line multi-angle light scattering, confirming that these proteins were dimers. The observed molecular masses extracted from the light scattering analyses are 92 kDa and 89 kDa, respectively (Fig. 1A), compared to the theoretical values of 46.1 kDa and 45.6 kDa, respectively, based on amino acid sequences. Both proteins gave rise to almost identical CD spectra with a predominantly α-helical signature (Fig. 1B).

### TRIM5α_rh_ CC-SPRY binds to HIV-1 CA and CA-NC tubular assemblies in a dose-dependent manner

**Figure 2:**
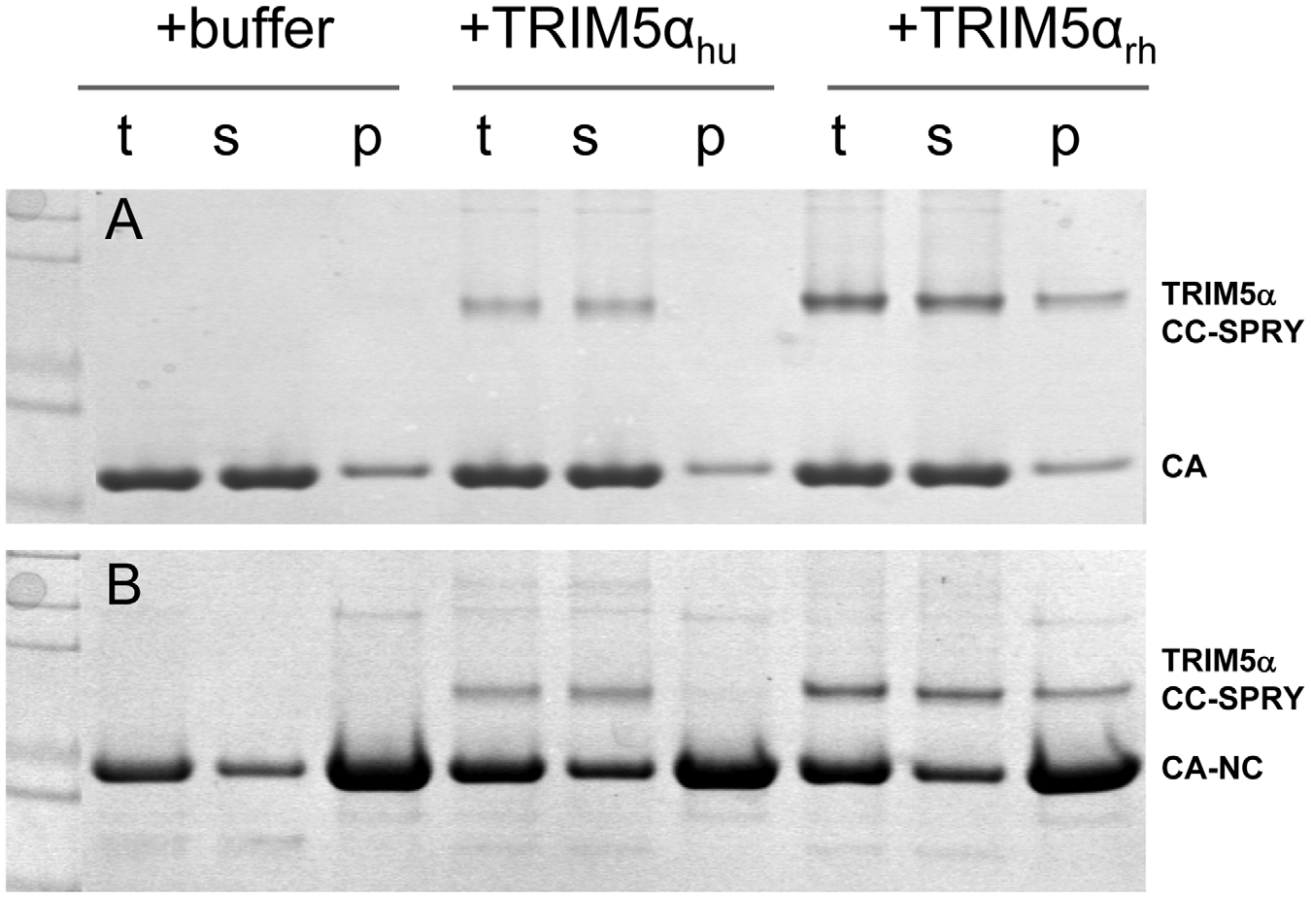
Binding of TRIM5α CC-SPRY to pre-assembled wild-type CA and CA-NC tubes. (A) SDS-PAGE analysis of binding reactions using CA tubular assemblies (64 µM), incubated with either TRIM5α_hu_ CC-SPRY (10 µM), TRIM5α_rh_ CC-SPRY (20 µM), or binding buffer. A control experiment under similar condition is shown in Fig. S1. (B) SDS-PAGE analysis of binding reactions using CA-NC tubes (2 mg/ml), incubated with either TRIM5α_hu_ CC-SPRY (10 µM), TRIM5α_rh_ CC-SPRY (20 µM) or binding buffer. Samples of the reaction mix before centrifugation (t), of supernatant (s), and of pellet (p) are shown.

**Figure 3:**
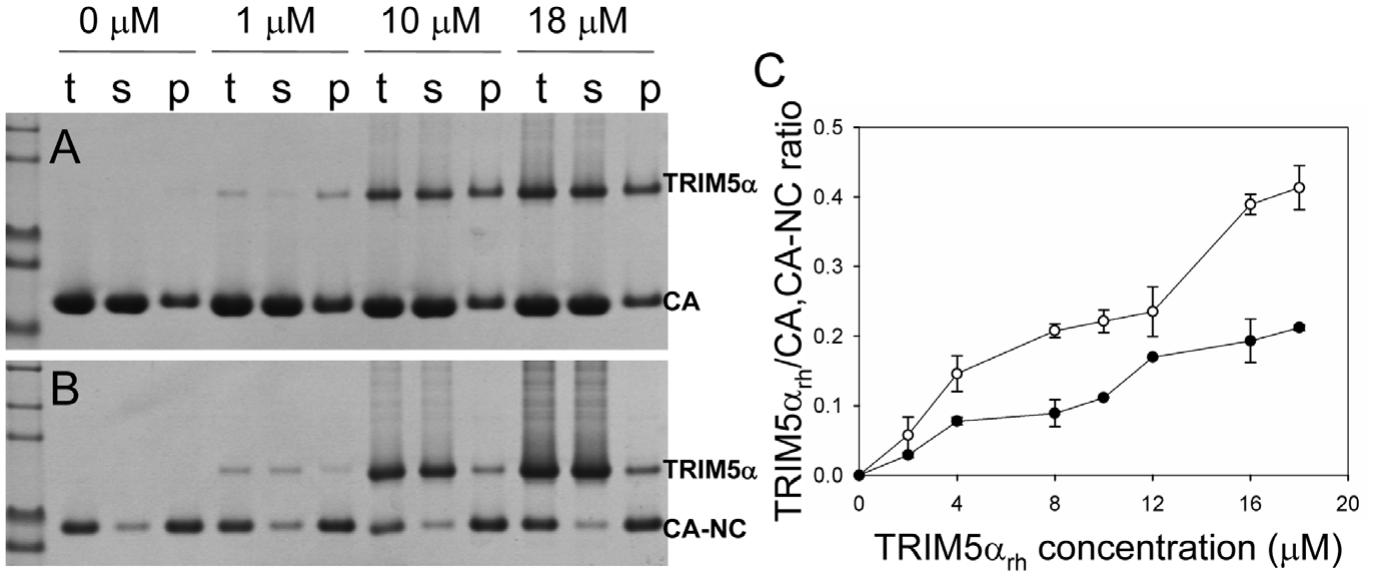
Analysis of TRIM5α_rh_ CC-SPRY binding to the assembly of wild-type CA and CA-NC tubes. (A&B) Increasing concentrations of TRIM5α_rh_ CC-SPRY (0, 1, 10, 18 µM) were incubated with CA tubular assemblies (64 µM) (A) or with CA-NC tubular assembly mixture (10 µM) (B) and analyzed by 10% SDS-PAGE. Samples of the reaction mix before centrifugation (t), of supernatant (s), and of pellet (p) are shown. (C) TRIM5α_rh_ CC-SPRY/CA (open circles) and TRIM5α_rh_ CC-SPRY/CA-NC (closed circles) binding ratios at the indicated input concentrations of TRIM5α_rh_ CC-SPRY. Molar ratios of CA- or CA-NC-bound TRIM5α were determined by gel densitometry of proteins stained with Coomassie Blue in the appropriate lanes of the SDS-PAGE gels. Three independent experiments were carried out in duplicates. Mean values (± s.d.) are plotted.

It is widely accepted that the restriction specificity of TRIM5α resides in its SPRY domain and that this domain interacts with retroviral capsids [1], [3], [11], [14], [15], [36]. However, only recently has direct binding been demonstrated for a TRIM5-21R fusion chimera with CA-NC assemblies [32], [34]. We used recombinant TRIM5α CC-SPRY proteins to examine direct binding to CA and CA-NC assemblies. Incubation of preassembled HIV-1 CA or CA-NC tubes with TRIM5α_rh_ resulted in co-sedimentation of TRIM5α_rh_ CC-SPRY/CA or TRIM5α_rh_ CC-SPRY/CA-NC complexes, respectively, in the pelleted fractions (Fig. 2, Fig. S1). More TRIM5α_rh_ was observed bound to CA assemblies than to CA-NC assemblies (Figs. 2 & 3). In contrast, we observed negligible binding of TRIM5α_hu_ CC-SPRY to HIV-1 CA or CA-NC complexes under the same assay conditions (Fig. 2). These data are consistent with previous results that demonstrated the inability of TRIM5α_hu_ to bind and restrict HIV-1, but a capacity for the same protein to recognize N-tropic murine leukemia virus (MLV) capsid [3], [4], [12].

A more quantitative analysis of TRIM5α_rh_ binding was carried out by measuring molar ratios of CA and CA-NC-bound TRIM5α_rh_ CC-SPRY over a range of TRIM5α_rh_ concentrations. Dose-dependent binding was observed for both CA and CA-NC assemblies (Fig. 3). Consistently, at all concentrations, TRIM5α_rh_ CC-SPRY bound CA more efficiently than CA-NC. This could be due to differences in CA and CA-NC structures on the surfaces of the assemblies, or differences in the flexibility of these assemblies, as CA-NC tubes were assembled in the presence of oligonucleotide. The binding ratios were 0.41 for TRIM5α_rh_ CC-SPRY/CA and 0.21 for TRIM5α_rh_ CC-SPRY/CA-NC, respectively, for the highest concentration of TRIM5α_rh_ CC-SPRY (18 µM). When a lower concentration of TRIM5α_rh_ CC-SPRY (1 µM) was used for binding to the CA-NC tubular assemblies (10 µM), a molar ratio of 0.034 was obtained. This ratio is somewhat lower than the value reported by Langelier et al. for TRIM5-21R by immunoblotting [32]. The lower binding ratio for TRIM5α_rh_ CC-SPRY is expected, since it lacks the self-associating B-box 2 domain, compared to the TRIM5-21R fusion protein. Furthermore, incubation with CC-SPRY_rh_ did not alter the fraction of pelletable CA and CA-NC, even at the highest TRIM5α_rh_ CC-SPRY concentrations (Figs. 2&3). These results are in accord with those reported for TRIM5-21R [32] and a binding study with CA-NC assemblies using TRIM5α_rh_-containing lysates [37]. Taken together, the data indicate that dimeric TRIM5α_rh_ CC-SPRY directly interacts with tubular CA and CA-NC assemblies and that binding of TRIM5α_rh_ does not dissociate these assemblies into soluble monomeric CA protein.

### Binding of TRIM5α_rh_ CC-SPRY to tubular CA assemblies releases discrete, linear fragments

**Figure 4:**
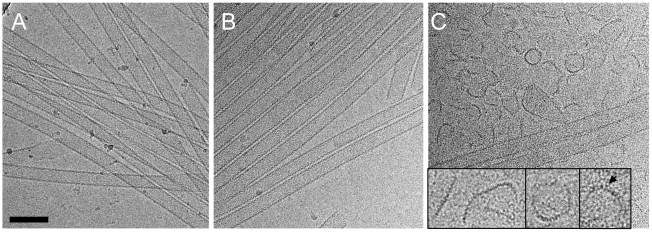
CryoEM analysis of the TRIM5α CC-SPRY interaction with wild-type CA tubes. (A–C) Low-dose projection images of CA assemblies (64 µM), incubated with binding buffer (A), human (B), or rhesus (C) TRIM5α CC-SPRY (18 µM). The displayed images are representative examples of four independent experiments. Inset, representative CA fragments, observed after TRIM5α_rh_ CC-SPRY binding. Arrows indicate the TRIM5α_rh_ CC-SPRY density on the CA fragments. Scale bars are 100 nm.

Although no dramatic effect of purified TRIM5α_rh_ on uncoating has been observed *in vitro* using CA-NC assemblies [32], a substantial decrease in intact CA-NC tubes was noted when TRIM5α_rh_-containing cell lysates were mixed with CA-NC tubular assemblies [37]. To investigate this apparent dichotomy, we carried out cryoEM structural analyses of the samples that were used in the TRIM5α CC-SPRY/CA tubular assembly binding assays (Figs. 2A&3A). CryoEM micrographs showed well-ordered CA tubular structures after incubation with binding buffer only (Fig. 4A) or TRIM5α_hu_ CC-SPRY (Fig. 4B), similar to our previously described assemblies [38]. In contrast, incubation of CA tubular assemblies with TRIM5α_rh_ CC-SPRY (18 µM) resulted in a massive structural break-down of the tubes (Fig. 4C), accompanied by the appearance of distinct fragments composed of strings of hexamers (Fig. 4C inset) [38]. The remaining tubes had generally lost the regularity of the hexagonal lattice. Some TRIM5α_rh_ CC-SPRY densities apparently remained on several of the fragments (Fig. 4C inset). Gold-labeling of TRIM5α_rh_ CC-SPRY in complex with CA tubular assemblies confirmed that TRIM5α_rh_ CC-SPRY bound to the CA assemblies (Fig. S2). These break-down fragments were primarily present in the pellet fraction after centrifugation (Fig. 3A), confirmed by cryoEM imaging of the pellet samples (Fig. S3), explaining why no effect on uncoating was detected in assays that measure soluble CA [32], [37]. These results suggest that the predominant effect of TRIM5α_rh_ is the break down of HIV-1 capsids into fragments and not the dissociation into soluble monomers.

We further examined the effect of CA mutations on TRIM5α_rh_ disruption. Several CA mutants, including A92E, which was used in our previous structural study [38], and the E45A mutant, which produces hyperstable capsids, were analyzed. The effect of TRIM5α_rh_ CC-SPRY binding to A92E CA tubular assemblies was similar to that observed with wild-type CA (Fig. S4A&B). The CA tubular assemblies carrying the capsid-stabilizing E45A mutation [46] also experienced structural damage by TRIM5α_rh_, but to a lesser degree (Fig. S4C&D). This suggests that the overall stability of HIV-1 capsid assemblies may modulate or interfere with TRIM5α_rh_ function, consistent with findings that hyperstable capsid core mutants effectively saturate TRIM5α-mediated restriction [5].

### Cross-linking of the inter-hexamer CA interface prevents TRIM5α_rh_ disruption

**Figure 5:**
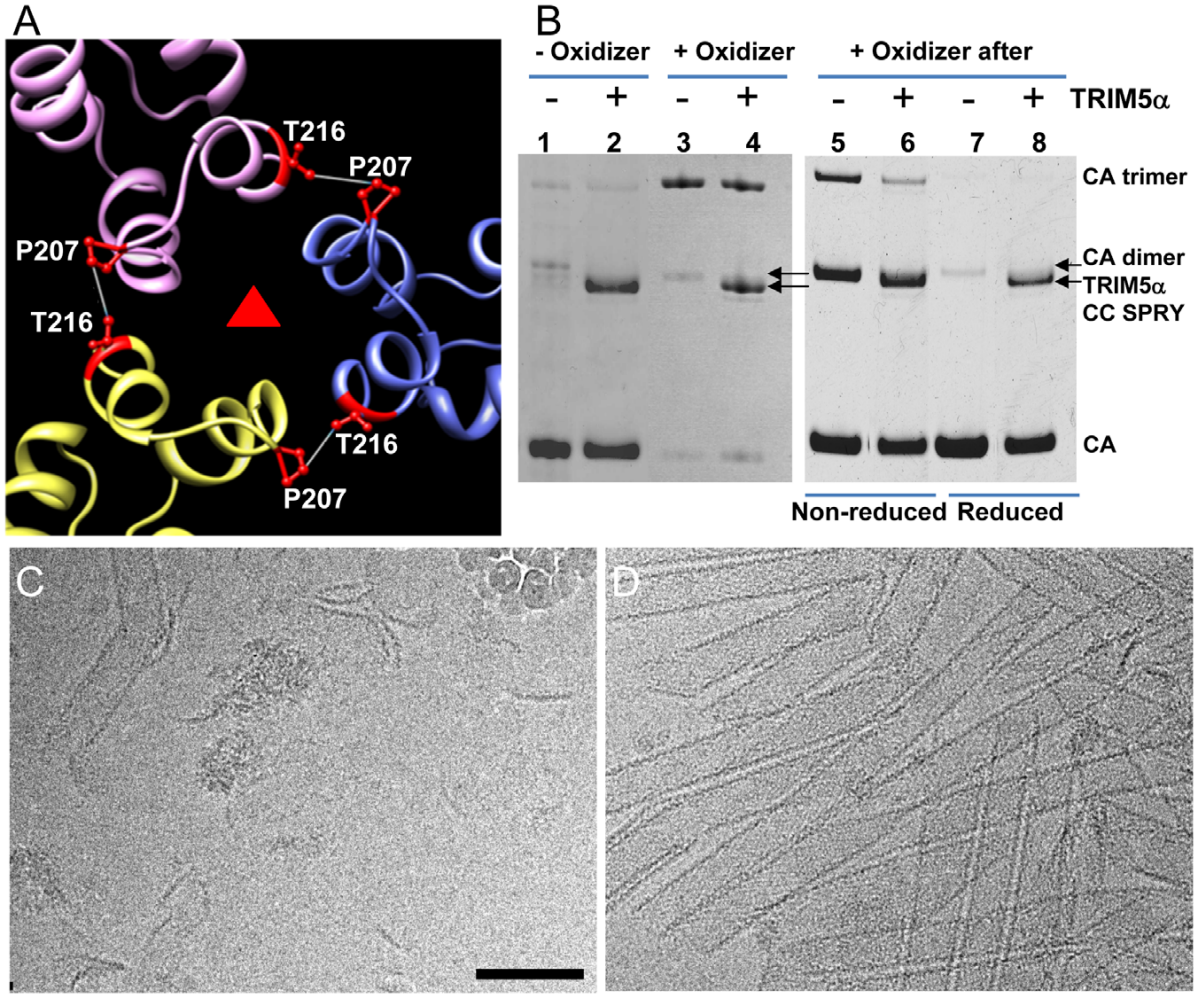
Cross-linked CA assemblies resist structural damage by TRIM5α_rh_ CC-SPRY. (A) Amino acids at the pseudo three-fold axis in the molecular model of the tubular CA assemblies [38] were used to guide cysteine mutagenesis (P207C/T216C) for cross-linking of CA tubes. (B) Non-reducing SDS-PAGE analysis of TRIM5α_rh_ CC-SPRY (18 µM) binding to cross-linked P207C/T216C CA tubes (left) and cross-linking of P207C/T216C CA tubes after TRIM5α_rh_ CC-SPRY (18 µM) binding (right), visualized by Coomassie Blue staining. Pellets of non-reduced and reduced samples were analyzed in lanes 5&6 and 7&8, respectively. (C&D) CryoEM analysis of the structural effect of TRIM5α_rh_ CC-SPRY binding to P207C/T216C CA tubes without (C, corresponding sample in panel B, lane2) and with cross-linking (D, corresponding sample in panel B, lane4). Some ice particles inadvertently deposited on the EM grid during cryo-sample preparation are visible in panel C (upper right-hand region). The displayed images are representative examples of three independent experiments. Scale bar, 100 nm.

To determine which interface in the capsid lattice is disrupted by CC-SPRY_rh_, we tested the effect of TRIM5α_rh_ CC-SPRY on cross-linked CA tubular assemblies. In previous work, we showed that introduction of a pair of cysteines, P207C/T216C, at the pseudo three-fold inter-hexamer interface, efficiently cross-linked three neighboring CA molecules into trimers upon oxidation (Fig. 5A&B). The interactions at this interface are mediated by the CA-CTD, predominantly helices H10 and H11 [38]. Such cross-linked P207C/T216C CA tubular assemblies are expected to contain stronger hexamer-hexamer interactions, stabilizing the lattice. The P207C/T216C mutant assembles into tubular structures very similar to the wild-type CA (Fig. S5). Both oxidized and non-oxidized P207C/T216C CA tubular assemblies bound TRIM5α_rh_ CC-SPRY, without any significant difference between them (Fig. 5B, lanes 1-4). However, cryoEM analysis revealed that TRIM5α_rh_ CC-SPRY exerted very little structural damage onto the cross-linked tubes, whereas the non-oxidized tubular assemblies exhibited similar structural breakdown as seen for wild type CA tubes (Fig. 5C&D). These data suggest that TRIM5α_rh_ CC-SPRY engages in inter-hexamer binding, most likely pulling apart the trimer interface, thereby disrupting the assembled tubes. We further tested this possibility by measuring the cross-linking efficiency of P207C/T216C CA assembly after TRIM5α_rh_ CC-SPRY treatment. As can be seen from the results illustrated in Fig. 5B (lanes 5&6), the level of cross-linked trimers was significantly reduced after incubation with TRIM5α_rh_ CC-SPRY. The percentage of the cross-linked CA trimer over total CA in the reduced sample is 3-fold less in the TRIM5α_rh_ CC-SPRY treated sample, compared to untreated sample, confirming that the trimer interface between three neighboring hexamers is disrupted by TRIM5α_rh_ CC-SPRY.

An alternative scenario could involve binding of the TRIM5α CC-SPRY dimer within a CA hexamer, with TRIM5α_rh_ CC-SPRY dimers pushing apart the hexamers. However, simple geometric considerations make this a very unlikely scenario if TRIM5α_rh_ SPRY binds near the cyclophilin A binding loop in CA [39], since the distance between two sites (>110 Å) is too large for the TRIM5α CC-SPRY dimer protein to span. Nonetheless, we tested for this possibility using a A14C/E45C CA double cysteine mutant, which can cross-link CA within hexamers [40]. Following incubation with TRIM5α_rh_ CC-SPRY, crosslinked A14C/E45C CA assemblies exhibited only a slight reduction in CA hexamers (Fig. S6, compare lanes 2 & 5), compared to the dramatic reduction of the trimer in the P207C/T216C CA assemblies (Fig. 5B, right panel). This small effect on the CA hexamer could be caused by minor perturbations at the intra-hexamer CA interfaces upon TRIM5α_rh_ CC-SPRY binding. Small amounts of CA dimer (∼50kD, Fig. S6, lanes 1, 3, 5, 7&9) in the non-oxidized assemblies and dimer of hexamers (∼280kDa, Fig. S6, lanes 2&8) in the oxidized A14C/E45C CA assemblies were observed by SDS-PAGE, possibly due to the CA CTD dimer interaction. Interestingly, the amount of hexamer dimers was greatly diminished in the TRIM5α_rh_ CC-SPRY treated sample (Fig. S6, lane 5 compared to lane 2&8). Again, these data further support that TRIM5α_rh_ CC-SPRY binding perturbs the CA inter-hexamer interface.

### TRIM5α_rh_ disrupts isolated HIV-1 cores similar to the in vitro capsid assemblies

**Figure 6:**
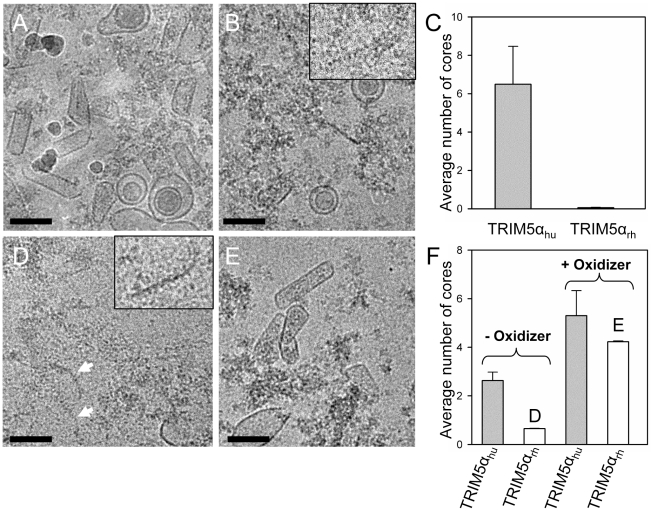
Effects of TRIM5α CC-SPRY_rh_ on isolated HIV-1 cores. (A&B) Low-dose projection images of purified mutant A14C/E45C cores (11 µg/ml) incubated with human (A) or rhesus (B) TRIM5α CC-SPRY (18 µM). Scale bars, 100 nm. Inset, enlarged view of a core fragment in the TRIM5α_rh_-treated sample. (C) Quantification of the number of cores on the cryoEM grids. The mean values of the average number of cores per image from four independent experiments (80 cryoEM images) are shown, with the error bars representing one standard deviation. (D&E) Representative low-dose projection images of purified P207C/T216C cores, incubated with rhesus TRIM5α CC-SPRY (18 µM), without (D) and with (E) oxidation for cross-linking. Scale bars, 100 nm. Inset, enlarged view of a core fragment in the TRIM5α_rh_-treated sample. (F) Quantification of the number of cores on the cryoEM grids. Representative cryoEM images from samples that are shown on panels D and E, respectively.

To extend the above *in vitro* studies to biological HIV-1 capsids, we examined the effect of TRIM5α_rh_ CC-SPRY on isolated HIV-1 cores. For this purpose, we purified cores from the HIV-1 CA mutants A14C/E45C and P207C/T216C for two reasons; first, the mutant cores appeared to be more stable through the isolation procedure, and second, A14C/E45C and P207C/T216C cores bear the same cysteine mutations that we used for the *in vitro* analysis described in the previous section. A14C/E45C and P207C/T216C cores were isolated from virions in high yield (average of 44% of virion-associated CA, vs. ∼15% typically observed for wild type) by brief detergent treatment and sucrose gradient sedimentation. The CA protein in A14C/E45C cores was readily cross-linked into hexamers, as shown by non-reducing SDS-PAGE analysis (Fig. S7). Despite the extensive CA hexameric crosslinking in A14C/E45C cores, incubation with TRIM5α_rh_ CC-SPRY resulted in a dramatic loss of intact cores observed by cryoEM, compared to the samples treated with the same amount of human TRIM5α CC-SPRY (Fig. 6A-C). In contrast, no significant reduction in the number of P207C/T216C cross-linked cores was seen upon TRIM5α_rh_ incubation (Fig. 6E and F, Fig. S7, +oxidizer samples). However, without ensuring effective cross-linking at the trimer interface (Fig. S7, -oxidizer), a four-fold decrease in the number of P207C/T216C cores was seen upon TRIM5α_rh_ treatment, compared to incubation with TRIM5α_hu_ (Fig. 6D and F, ­oxidizer samples). Although very few, a small number of P207C/T216C cores were observed in TRIM5α_rh_ treated samples, presumably due to low levels of spontaneous crosslinking of isolated P207C/T216C cores at the trimer interface. Furthermore, similar protofilament fragments as seen for the *in vitro* assemblies were also observed after TRIM5α_rh_ treatment of cores (Fig. 6D, arrows and inset). The above data demonstrate, for the first time, that TRIM5α_rh_ CC-SPRY is capable of exerting direct structural damage on the isolated HIV-1 cores and TRIM5α_rh_ binding preferentially disrupts the inter-hexamer interfaces in the HIV-1 capsid.

### A model for TRIM5α_rh_ action on HIV-1 capsid

**Figure 7:**
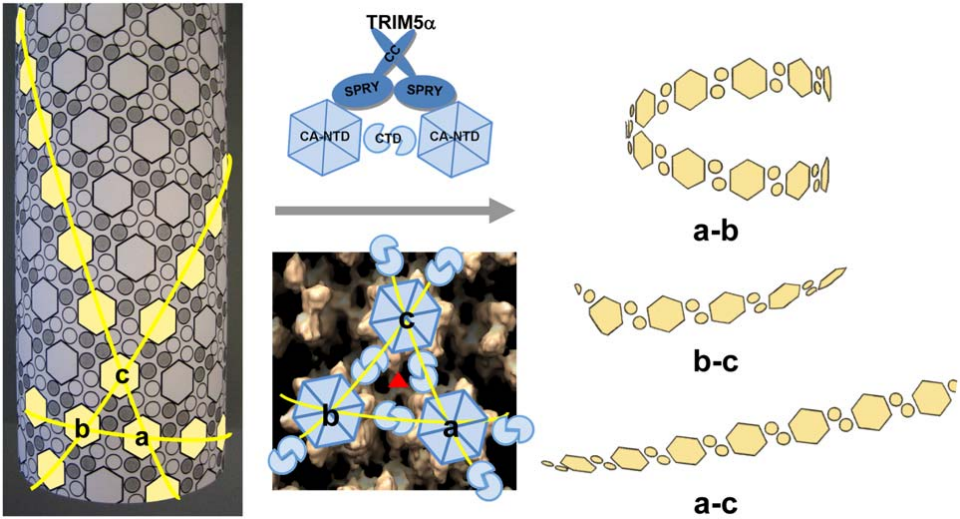
Model of TRIM5α_rh_ CC-SPRY in HIV-1 CA restriction. A schematic representation of the CA tubular assembly is shown at the left. CA NTD hexamers on the outside surface of the tube are depicted as hexagons, forming an extended hexagonal surface lattice that is connected by CA CTD dimers on the inner surface of the tube. Binding of TRIM5α CC-SPRY to assembled HIV-1 CA imposes stress on inter-hexamer interactions (middle panel, top) and weakens the CTD trimer interface (red triangle in middle panel, bottom), thereby causing damage to the lattice and releasing fragmented protofilaments. Based on our structural model for the CA tubular assemblies [38], three types of fragments containing linear arrays of CA hexameric units can be generated, depending on which of the three inter-hexamer interfaces are disrupted. For the short pitch helical arrays along the “a–b” direction, tightly curved or circular fragments are expected (top right), whereas significantly less curved fragments (bottom right) are expected for the longest pitch helical arrays along the “a–c” direction. Predicted fragments along the “b–c” direction should also be more linear than the tightly curved “a–b” direction fragments. Intermolecular cross-linking of the tubes strengthens the interfaces between the hexamers a, b and c, reducing TRIM5α_rh_ CC-SPRY-mediated destructive effects.

Examination of the fragments present in the cryoEM images revealed predominantly curved linear structures (Fig. 4C). These structures resemble fragments of protofilaments in CA helical assemblies. Our results are consistent with previous studies that TRIM5α_rh_ binding to CA-NC assemblies did not increase soluble CA-NC monomers and dimers [32], [37], and further suggest that binding of TRIM5α_rh_ disrupts the hexamer-hexamer interfaces, thereby releasing protofilaments along one of the three principal helical directions. A model based on the above findings is depicted schematically in Figure 7. CA assembles into helical tubes *in vitro* with a hexagonal surface unit formed by CA NTDs that is connected by CTD-CTD dimer and trimer interfaces on the inner surfaces of the three-dimensional tube or cone [38], [40], [41]. In these helical tubes, three slightly different inter-hexamer interactions were observed (see Fig. 3 in [38]). Binding of TRIM5α_rh_ may disrupt these interactions differentially, weakening the CTD-CTD interfaces between hexamers. In turn, this causes release of CA protofilament fragments, such as those illustrated in Figure 7, and, indeed, similar types of fragments were observed in the cryoEM images (Fig. 4C). For TRIM5-21R interacting with CA-NC, shortening of tubes was observed *in vitro,* in addition to fragmentation [32]. Examples of this type of fragmentation of helical tubes have also been observed in other biological systems, including microtubules *in vivo* and *in vitro* [42], [43], actin filaments [44] and dynamin spirals and tubes [45]. Thus, the disassembly of the CA tubes into helical-type fragments is not unprecedented. Importantly, the use of two mutants, A14C/E45C and P207C/T216C, containing engineered disulfide bonds, allowed us to assign the site of TRIM5α_rh_ action to the inter-hexamer interface (vs. the intra-hexamer interface), both, for *in vitro* assemblies and isolated HIV-1 cores, providing compelling evidence for specific structural disruption of the trimer interface of the HIV-1 capsid upon TRIM5α binding. In this manner, key insights into the mechanistic aspects of TRIM5α_rh_ - capsid interaction were obtained.

Retroviral uncoating is a poorly characterized process, generally defined as viral capsid disassembly following release of the viral core into the target cell. Studies using HIV-1 CA mutants indicate that the stability of the viral core is optimally balanced for efficient viral replication [46]. Therefore, a plausible mechanism for restriction by TRIM5α involves binding to the viral capsid, capsid destabilization, and perturbation of uncoating. Here, we show by cryoEM that TRIM5α_rh_ CC-SPRY binding to CA assemblies causes massive destruction of assembled HIV-1 CA complexes. A similar effect was observed with purified HIV-1 cores. Intriguingly, this effect was seen with the TRIM5α_rh_ CC-SPRY domain construct lacking the RING and B-box domains, albeit at high concentrations, even though TRIM5α protein devoid of RING and B-box domains was reported to lack restriction activity when expressed in cells [23], [24]. These seemingly inconsistent results could be due to several factors, including: (1) reduced binding to the viral capsid in the cell due to lack of self-association mediated by the B-box that can be overcome at high protein concentration *in vitro*; (2) improper intracellular localization of the deletion protein; or (3) altered association with host cell factors. We favor the first explanation, since the CC-CypA protein has been shown to restrict HIV-1 and FIV when expressed in target cells [47], and oligomerization of CypA appears sufficient to induce HIV-1 restriction [48]. Given the ability of the B-box domain to promote higher-order TRIM5α association [21], it seems plausible that this domain in intact TRIM5α_rh_ may potentiate the effects observed here for TRIM5α_rh_ CC-SPRY. Most importantly, while the CC-SPRY from rhesus TRIM5α was active on our *in vitro* assemblies and isolated cores, the corresponding human TRIM5α fragment was inactive. Thus, binding of the CC-SPRY domain to CA is essential for TRIM5α retroviral restriction and for structural disruption of the capsid. However, our current results do not exclude the possibility of additional structural consequences induced by higher-ordered oligomerization of TRIM5α on the viral capsid.

Although the molecular mechanism of TRIM5α restriction is not fully understood, current models hypothesize that after capsid release into the target cell, TRIM5α binds and triggers premature capsid disassembly. Our results suggest that direct binding of TRIM5α to the capsid is sufficient to inflict direct structural damage. Yet, cellular proteasome activity is clearly involved in the block to reverse transcription induced by TRIM5α[27]. Recruitment of proteasomes, most likely via the TRIM5α RING domain, may further disaggregate capsid fragments and also degrade TRIM5α [28], thereby mediating the irreversible block to infection. In contrast to TRIM5α-mediated restriction, Fv1 restriction of MLV does not result in inhibition of reverse transcription, yet both TRIM5α and Fv1 target the retroviral capsid. We speculate that the common feature in TRIM5α and Fv1 restriction is the structural damage to the capsid, with the major mechanistic difference involving recruitment of the proteasome in the case of TRIM5α-dependent restriction.

The findings presented here represent the first detailed structural analysis of TRIM5α disruption of the CA lattice to date. Additional structural studies of TRIM5α effects, especially with regard to the CTD-CTD interfaces in CA assemblies and HIV-1 cores, as well as the involvement of the RING and B-box domains, will further aid to elucidate the molecular mechanisms of TRIM5-mediated HIV-1 restriction and may offer insights into the HIV-1 virus-cellular interplay as well as lead to novel approaches in antiviral therapy.

## Materials and Methods

### Protein expression and purification

cDNAs encoding the coiled-coil and SPRY domains of human and rhesus TRIM5α (TRIM5α CC-SPRY; residues 132-493 and 134-497, respectively) were amplified and cloned into the pENT-TOPO vectors (Invitrogen), modified to encode a Strep-tag at the N-terminus and a His_6_-tag at the C-terminus of the proteins. The cDNAs encoding HIV-1 capsid (CA) and capsid- nucleocapsid (CA-NC) were amplified from pNL4-3 and cloned into the pET21 vector (Invitrogen). All clones were verified by sequencing of the entire coding region.

Baculoviruses expressing human and rhesus TRIM5α CC-SPRY were prepared using the Baculdirect C-term (Invitrogen) according to the manufacturer's protocols. Proteins were expressed in SF21 insect cells by infecting cells with recombinant baculoviruses at a MOI of 2 for 40 h. Cells were lysed by sonication in a buffer containing 25 mM sodium phosphate, pH 7.5, 250 mM NaCl, 10 mM beta-mercaptoethanol, and 0.02% sodium azide. Soluble proteins were purified over a 5 mL Ni-NTA column followed by passage over a Hi-Load Superdex 200 16/60 column (GE Healthcare) in a buffer containing 25 mM sodium phosphate, pH 7.5, 150 mM NaCl, 2 mM DTT, 10% glycerol, and 0.02% sodium azide. The fraction containing TRIM5α CC-SPRY was further purified over a 5 mL Hi-Trap QP column (GE Healthcare) using a gradient of 0–1 M NaCl or a 5 mL StrepTrap-HP column (GE-Healthcare) using 2.5 mM desbiotin for elution. CA-NC proteins were expressed in *E. coli* Rosetta 2 (DE3), cultured in Luria-Bertani medium, using 0.4 mM IPTG for induction and growth at 18°C for 23 h. The proteins were purified as described in Ganser et al [49]. Briefly, soluble proteins were precipitated with 40% (w/v) ammonium sulfate after DNA was removed by precipitation with polyethylenimine. The precipitates were dialyzed against a buffer containing 25 mM TrisHCl, pH 7.5, 50 mM NaCl, 1 µM ZnSO_4_, 10 mM beta-mercaptoethanol, and 0.02% azide. Proteins were separated by column chromatography over a 5 mL Hi-Trap SP (GE Healthcare) with a 0–1 M NaCl gradient and Hi-Load Superdex75 26/60 columns, equilibrated with a buffer containing 25 mM TrisHCl, pH 7.5, 150 mM NaCl, 1 µM ZnSO_4_, 10 mM beta-mercaptoethanol, and 0.02% azide. CA proteins were prepared as described in Byeon et al [38].

### Isolation of HIV-1 core structures

HIV-1 cores were isolated from virions by a modification of the “spin-thru” method previously described [50]. HIV-1 viruses were derived from the R9 molecular clone [51] and mutants thereof. CA mutations were created by overlap PCR. SpeI-ApaI fragments were transferred into R9, and the transferred region was verified by PCR. HIV-1 viruses were produced by transient transfection of sixty dishes of 6×10^6^ 293T cells with 10 µg plasmid DNA (using 10 µg of HIV-1 construct R9, R9.Env-, or R9.A14C/E45C) using polyethylenimine (3.6 µg/ml, Polysciences) [52] in each 10 cm dish. Two days after transfection, virus-containing supernatants were collected and clarified by filtration (0.45 µm pore-size). Particles in clarified supernatants (600 ml) from 293T cells were pelleted through 3ml cushions of 20% sucrose (120,000 ×*g*, 2.5 h) in a Beckman SW32Ti rotor then gently suspended in a total of 1.2 ml STE buffer (10 mM Tris-HCl [pH 8.0], 100 mM NaCl, 1 mM EDTA) for 2 h at 4°C. The concentrated virus suspension was subjected to equilibrium ultracentrifugation (120,000 × *g*, 16 h, 4°C, Beckman SW-32Ti rotor) through a layer of 1% Triton X-100 into a linear gradient of 30%–70% sucrose in STE buffer. Twelve 1-ml fractions were collected. CA concentrations were determined by p24 ELISA [53]. The peak p24 fractions near the bottom of the gradient were pooled and concentrated to ∼100 µl by diafiltration with an Ultracel-10K protein concentrator (Amicon). The sample was diluted with STE buffer and reconcentrated to reduce the final sucrose concentration in the sample to less than 0.5%. The concentrated samples of cores were then assayed for p24 by ELISA.

### Multi-angle light scattering

Light-scattering data were obtained using an analytical Superdex200 column (1 cm ×30 cm, GE Healthcare) with in-line multi-angle light scattering (HELEOS, Wyatt Technology), variable wavelength UV (Agilent 1100 Series, Agilent Technology) and refractive index (Optilab rEX, Wyatt Technology.) detection. Approximately 100 µL of 2 mg/mL protein solutions were injected into the pre-equilibrated column using 25 mM sodium phosphate buffer (pH 7.5), 250 mM NaCl, 10% glycerol, and 0.02% (w/v) sodium azide at a flow-rate of 0.5 ml/minute for equilibration and elution. Molecular masses were determined from the scattering data using the ASTRA program (Wyatt Technology).

### Circular Dichroism (CD)

CD spectra of TRIM5α CC-SPRY (5.4 µg/mL) were collected in a buffer containing 1 mM sodium phosphate, pH 7.5, 14 mM NaCl with a Jasco-810 CD spectrophotometer (Easton, MD). Data were collected with a scan rate of 1 nm/sec from 260 to 200 nm at a constant temperature of 12°C and averaged over 40 scans.

### Binding assays

CA and CA-NC tubes were assembled containing 80 µM (2 mg/ml) CA, 1 M NaCl and 50 mM Tris-HCl (pH 8.0) at 37°C for one hour or 300 µM CA-NC, 60 µM TG50 oligonucleotide in 250 µM NaCl, 50 mM Tris-HCl (pH 8.0) buffer at 4°C for 19 hr, respectively. For the TRIM5α CC-SPRY binding assays, the binding buffer, 10 mM Tris pH 7.5, 330 mM NaCl, 1 mM TCEP, 0.02% Azide, 5% Glycerol, is also the stock buffer for TRIM5α CC-SPRY proteins. Briefly, binding buffer containing different concentrations of TRIM5α CC-SPRY was added to preassembled CA and CA-NC tubes. CA concentration was slightly reduced to 64 µM in the binding assays. The CA-NC assemblies were diluted to final concentrations of 80 µM (comparable to the amount of total protein used with CA) or 10 µM (comparable to the number of tubes seen with CA) with assembly buffer prior to the binding assays. TRIM5α_hu_CC-SPRY or TRIM5α_rh_CC-SPRY aliquots from 4 mg/ml stock solutions were added to preassembled CA and CA-NC tubes. The reaction mixture was incubated on a rocking platform at room temperature for 1 hr with gentle mixing at 10 min intervals. At the end of incubation, 5 µl samples were withdrawn from the reaction mixtures and immediately used for cryoEM analysis. 6 µl samples from the same reaction mixtures were mixed with 4X LDS loading buffer (Invitrogen) supplemented with 10 mM DTT for SDS-PAGE analysis (t). The remaining sample was pelleted at 20,000 g with an Eppendorf centrifuge 5417R for 15 min and supernatants (s) and pellets (p, resuspended in 1/3 of volume) were mixed with 4X LDS loading buffer for gel analysis. Total, supernatant, and pellet samples, without boiling, were loaded on 10% SDS-PAGE and stained with Coomassie Blue. Each experiment was carried out at least three times.

### Gold labeling of TRIM5α CC-SPRY

His-tagged TRIM5α proteins at the C-terminus, TRIM5α_hu_CC-SPRY and TRIM5α_rh_CC-SPRY, were labeled using 5 nm Ni-NTA-Nanogold gold beads from Nanoprobes (Yaphank, NY). For gold labeling, wild type CA protein was assembled into tubes using 80 µM (2 mg/ml) CA in the assembly buffer (1 M NaCl and 50 mM Tris-HCl (pH 8.0)) at 37°C for one hour. TRIM5α_hu_CC-SPRY or TRIM5α_rh_CC-SPRY (2 µl) was added to the assembly mix (20 µl) to a final concentration of 18 µM and incubated on a rocking platform at room temperature for 1 hr with gentle mixing at 10 min intervals. 2.7 µl of 5 nm Ni-NTA-Nanogold gold beads (stock concentration, 0.5 µM) in 100 mM imidazole (pH 8.0) was added to the assemblies and allowed to incubate at room temperature for 20 minutes. The mixture was then centrifuged at 3,000 g and the pellet was resuspended in assembly buffer. Samples were immediately applied to glow-discharged EM grids for negative staining with 1% uranyl acetate solution after resuspension. Images were acquired on an FEI Tecnai TF20 electron microscope at a nominal magnification of 50,000 and with underfocus values about 2 µm, using a Gatan ultrascan 4KX4K CCD camera (Gatan Inc., Pleasanton, CA, U.S.A.).

### CA double-cysteine mutant cross-linking reactions

The cross-linking experiment was set up as previously described [54]. Briefly, 30 µl P207C/T216C or A14C/E45C CA were preassembled in the presence of 50 µM DTT under the conditions described above. The assembled material was then subjected to centrifugation at 20,000 g at room temperature in an Eppendorf centrifuge 5417R for 15 minutes. The pellet was resuspended in 30 µl assembling buffer and oxidized with 1 µl of 30x oxidizer mix (60 µM CuSO_4_, (Sigma) dissolved in water, and 267 µM 1,10-Phenanthroline (Sigma) dissolved in 100% ethanol in a 1∶1 ratio) for 5 seconds, immediately followed by quenching with 20 mM iodoacetamide (Sigma) and 3.7 mM Neocuproine (Sigma).

### SDS-PAGE gel densitometry analysis

For the dose-dependent TRIM5α_rh_ CC-SPRY binding assay, the SDS-PAGE gels were scanned (Epson 4990 scanner) and the integrated intensities of CA, CA-NC, and TRIM5α_rh_ protein bands in pellet fractions were measured using Image J 1.40 g program (NIH). The molar ratios were calculated according to the formula (TRIM5α_rh_ band intensity/TRIM5α_rh_ molecular weight)/(CA band intensity/CA molecular weight).

### Cryo-EM analysis

Aliquots from the binding assays (above) were subjected to cryoEM analysis. 2 µl were applied to the carbon side of a glow discharged perforated Quantifoil grids (Quantifoil Micro Tools, Jena, Germany), and 2.5 µl binding buffer was added to the back side of the grids. Grids were blotted and plunge-frozen in liquid ethane using a manual gravity plunger. Low dose (10∼15 e^−^/Å^2^) projection images were collected on an FEI Tecnai TF20 electron microscope at a nominal magnification of 50,000 and with underfocus values ranging from 1.0 to 2.5 µm, using a Gatan ultrascan 4KX4K CCD camera (Gatan Inc., Pleasanton, CA, U.S.A.).

### Quantification of A14C/E45C and P207C/T216C cores in the presence of human and rhesus TRIM5α CC-SPRY

The effect of Rhesus TRIM5α CC-SPRY on HIV-1 cores was examined and quantified using cryoEM. 18 µM rhesus or human TRIM5α CC-SPRY proteins were added to a solution of isolated HIV-1 A14C/E45C or P207C/T216C cores (∼11 µg/ml). After one hour incubation at room temperature with gentle agitation, the samples were subjected to cryoEM analysis. For each sample, about 80 low dose projection images were collected at 19,000x magnification. Each field of view covers about 5 µm^2^. The image areas were chosen randomly, owing to the nature of cryoEM imaging. The number of cores in each sample was quantified using average number of cores per image frame. Mean values from four totally independent experiments are plotted in Fig. 6 with the standard deviation indicated.

## Supporting Information

Figure S1 SDS-PAGE analysis of binding of TRIM5α CC-SPRY to pre-assembled wild-type CA tubes. Samples of the reaction mix before centrifugation (t), of supernatant (s), and of pellet (p) are shown. Controls for TRIM5α without CA, CA without TRIM5α and CA with human TRIM5α are shown as indicated.
(TIF)

Figure S2 Gold labeling TRIM5α CC-SPRY. A) CA tubular assemblies incubated with TRIM5α_hu_CC-SPRY. (B&C) CA tubular assemblies incubated with TRIM5α_rh_CC-SPRY. (D) A gallery of gold-labeled TRIM5α_rh_CC-SPRY in complex with CA tubes. Scale bars, 100 nm.
(TIF)

Figure S3 CryoEM micrographs of supernatant (A&B) and pellet (C&D) fractions of the TRIM5α_rh_ CC-SPRY/CA mixture at low magnification (3,000x, A&C) and high magnification (50,000X, B&D). CA fragments appear in the pellet fraction after centrifugation. Scale bars, 2 um in A&C and 100 nm in B&D.
(TIF)

Figure S4 Low dose projection images of CA mutant assemblies treated with rhesus TRIM5α CC-SPRY. (A-B) Comparison of A92E CA assemblies treated with 0 µM (A) or 18 µM (B) of TRIM5α_rh_ CC-SPRY. Fragmented CA helical arrays similar to those in observed in the wild-type CA samples are seen. Binding of TRIM5α_rh_ CC-SPRY also causes bundling of A92E tubes (indicated by arrows in panel B). (C&D) Comparison of E45A CA assemblies treated with 0 µM (A) or 18 μM (B) of TRIM5α_rh_ CC-SPRY. Fewer fragments were observed compared to wild-type CA and A92E CA. Scale bars, 100 nm.
(TIF)

Figure S5 (A) HIV-1 P207C/T216C CA can efficiently assemble into short tubes. (B) Addition of oxidizer to the assembly solution does not introduce any noticeable structural changes in the P207C/T216C CA tubes. Scale bars, 100 nm.
(TIF)

Figure S6 SDS-PAGE analysis of TRIM5α_rh_ CC-SPRY binding to A14C/E45C CA tubes. A14C/E45C CA assemblies were incubated with either TRIM5α_hu_ CC-SPRY, TRIM5α_rh_ CC-SPRY or reaction buffer followed by oxidization. Samples of the reaction mixture before high speed centrifugation (t), and pellets of non-reduced (p) and reduced (pDTT) samples were analyzed by non-reducing SDS-PAGE and stained with Coomassie Blue. A CA dimer is observed (lane 1, 3, 5, 7, 9) in non-oxidized samples, whereas a dimer of CA hexamers is only seen in oxidized A14C/E45C CA assemblies without TRIM5α_rh_ CC-SPRY treatment (lane 2 and 8).
(TIF)

Figure S7 Non-reducing SDS-PAGE analysis of isolated HIV-1 A14C/E45C and P207C/T216C cores, detected by immunoblotting with rabbit anti-CA serum. Lane 1, A CA oligomer ladder formed by purified, cross-linked P17C/T19C CA (gift from Dr. Owen Pornillos [Pornillos O, Ganser-Pornillos BK, Banumathi S, Hua Y, & Yeager M (2010) Disulfide bond stabilization of the hexameric capsomer of human immunodeficiency virus. J Mol Biol 401(5):985-995.]); lane 2, isolated A14C/E45C cores contained predominantly hexameric CA; lane 3, isolated P207/T216C cores contained primarily monomeric CA; lane 4, isolated P207/T216C cores contained primarily CA trimers after oxidization; lane 5, molecular weight markers.
(TIF)
