# Supplementary figures and images for: Rhesus TRIM5α Disrupts the HIV-1 Capsid at the Inter­Hexamer Interfaces
Source: PLoS Pathog. 2011 Mar 24;7(3):e1002009. doi: 10.1371/journal.ppat.1002009 (PMC3063768; doi:10.1371/journal.ppat.1002009)

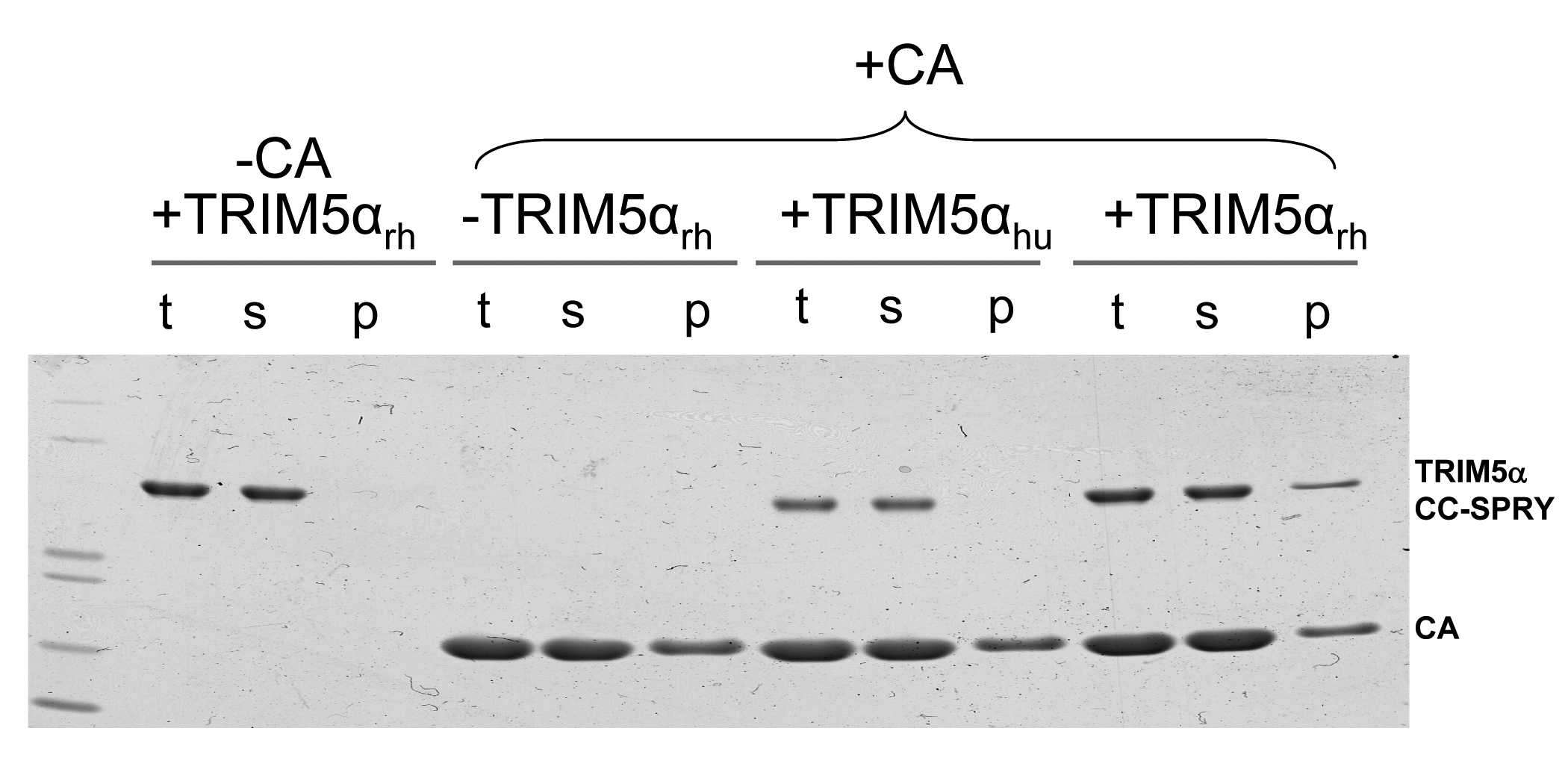

Supplement: Figure S1 — SDS-PAGE analysis of binding of TRIM5α CC-SPRY to pre-assembled wild-type CA tubes. Samples of the reaction mix before centrifugation (t), of supernatant (s), and of pellet (p) are shown. Controls for TRIM5α without CA, CA without TRIM5α and CA with human TRIM5α are shown as indicated. (TIF) [file ppat.1002009.s001.tif]

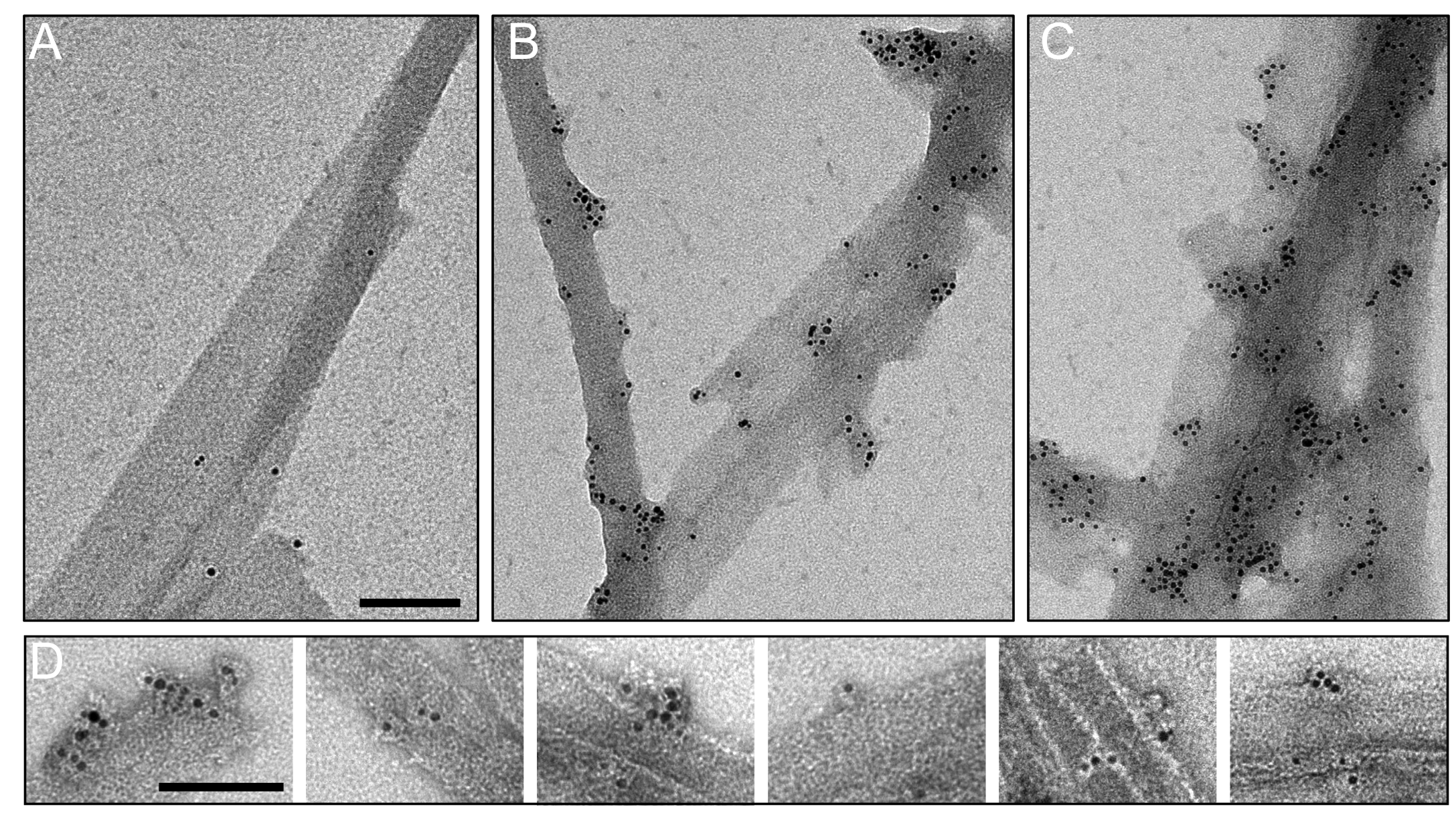

Supplement: Figure S2 — Gold labeling TRIM5α CC-SPRY. A) CA tubular assemblies incubated with TRIM5αhuCC-SPRY. (B&C) CA tubular assemblies incubated with TRIM5αrhCC-SPRY. (D) A gallery of gold-labeled TRIM5αrhCC-SPRY in complex with CA tubes. Scale bars, 100 nm. (TIF) [file ppat.1002009.s002.tif]

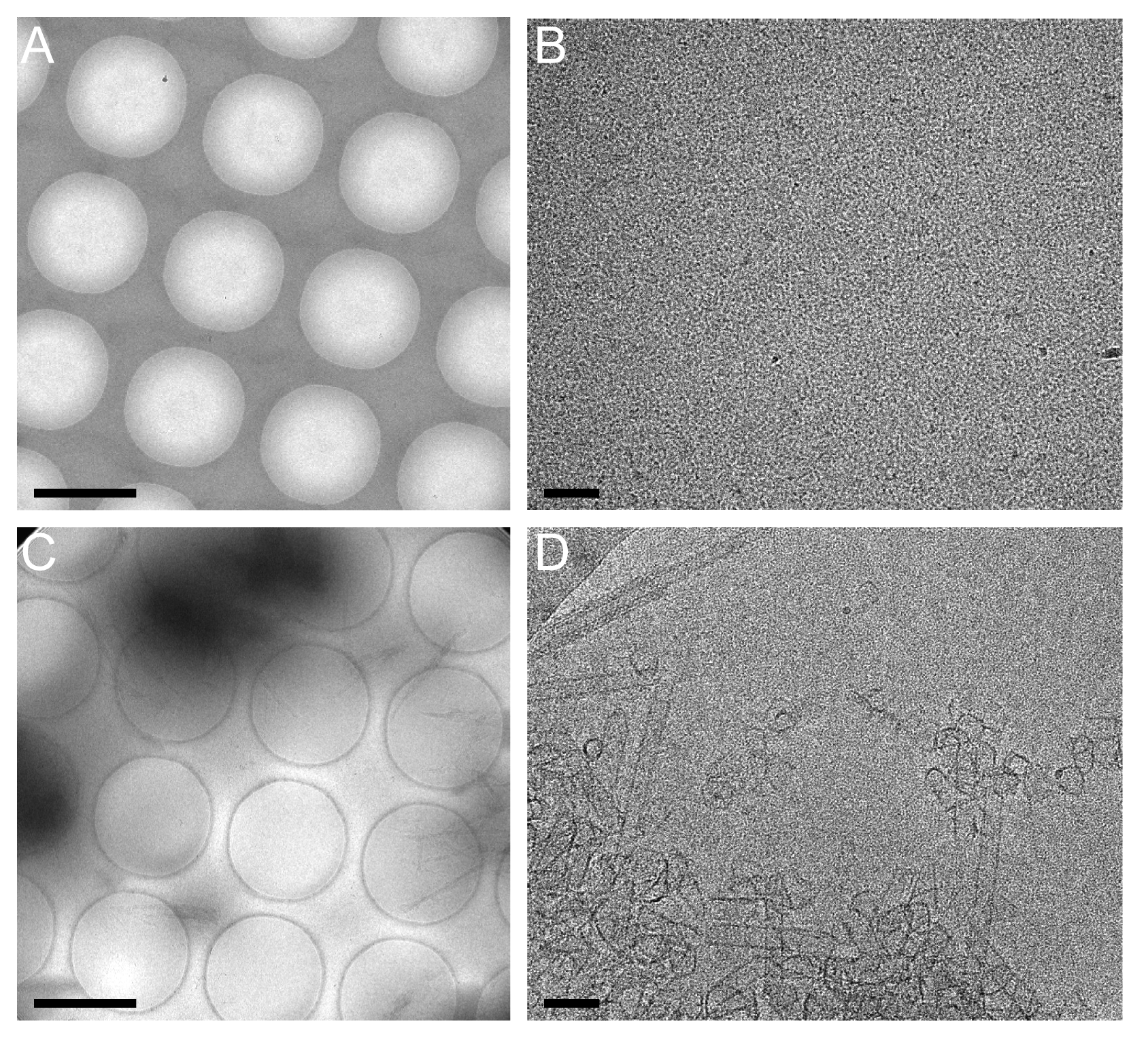

Supplement: Figure S3 — CryoEM micrographs of supernatant (A&B) and pellet (C&D) fractions of the TRIM5αrh CC-SPRY/CA mixture at low magnification (3,000x, A&C) and high magnification (50,000X, B&D). CA fragments appear in the pellet fraction after centrifugation. Scale bars, 2 um in A&C and 100 nm in B&D. (TIF) [file ppat.1002009.s003.tif]

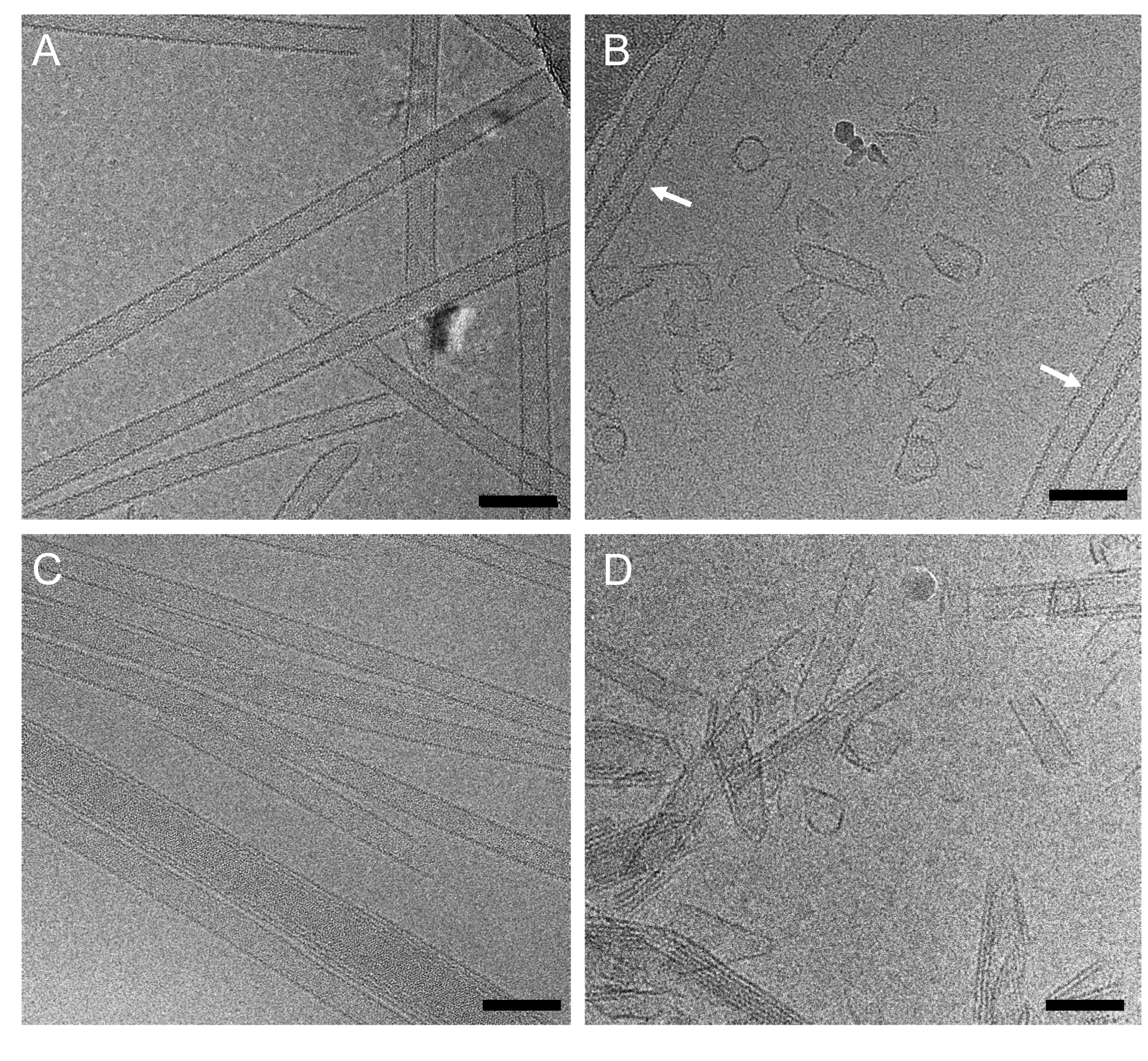

Supplement: Figure S4 — Low dose projection images of CA mutant assemblies treated with rhesus TRIM5α CC-SPRY. (A-B) Comparison of A92E CA assemblies treated with 0 µM (A) or 18 µM (B) of TRIM5αrh CC-SPRY. Fragmented CA helical arrays similar to those in observed in the wild-type CA samples are seen. Binding of TRIM5αrh CC-SPRY also causes bundling of A92E tubes (indicated by arrows in panel B). (C&D) Comparison of E45A CA assemblies treated with 0 µM (A) or 18 μM (B) of TRIM5αrh CC-SPRY. Fewer fragments were observed compared to wild-type CA and A92E CA. Scale bars, 100 nm. (TIF) [file ppat.1002009.s004.tif]

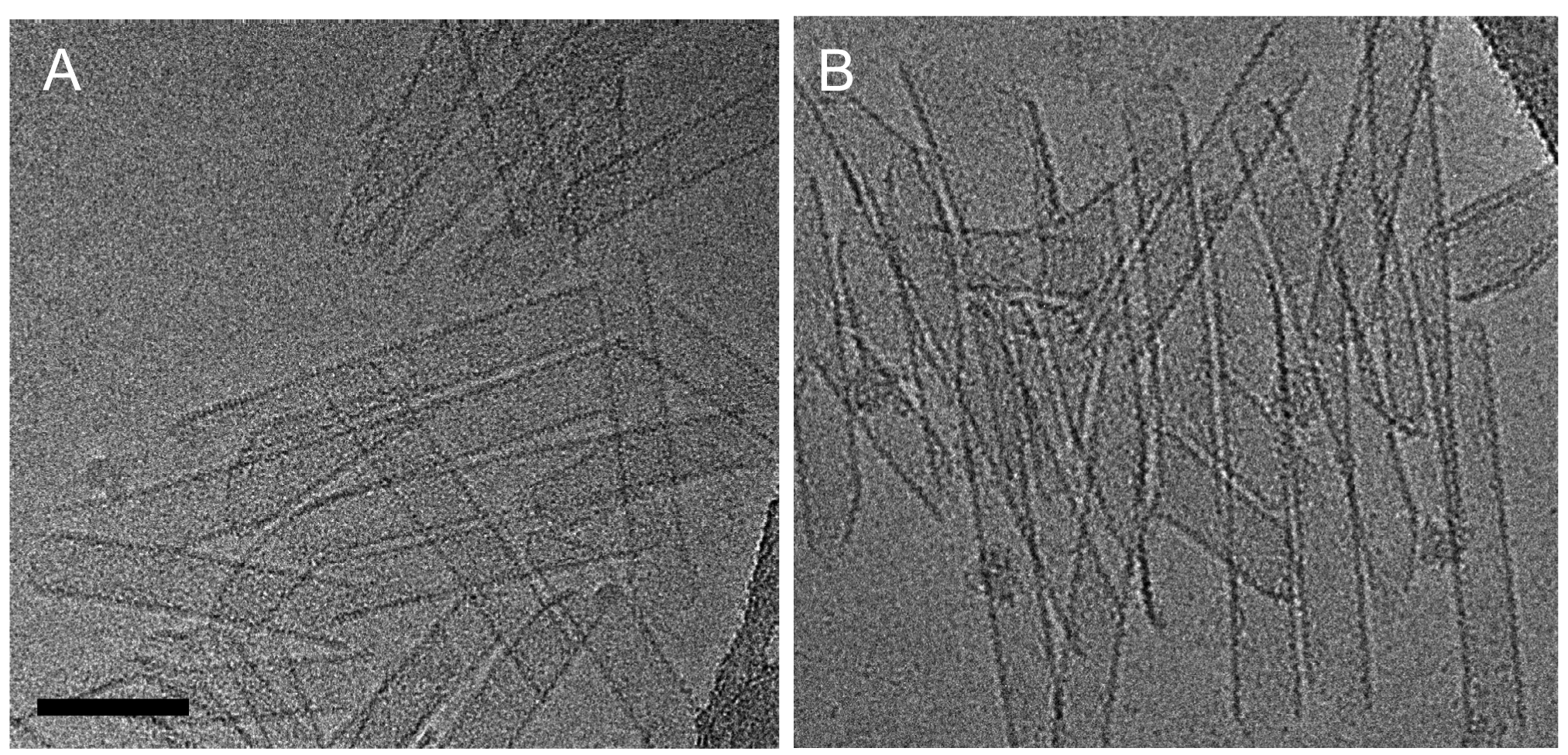

Supplement: Figure S5 — (A) HIV-1 P207C/T216C CA can efficiently assemble into short tubes. (B) Addition of oxidizer to the assembly solution does not introduce any noticeable structural changes in the P207C/T216C CA tubes. Scale bars, 100 nm. (TIF) [file ppat.1002009.s005.tif]

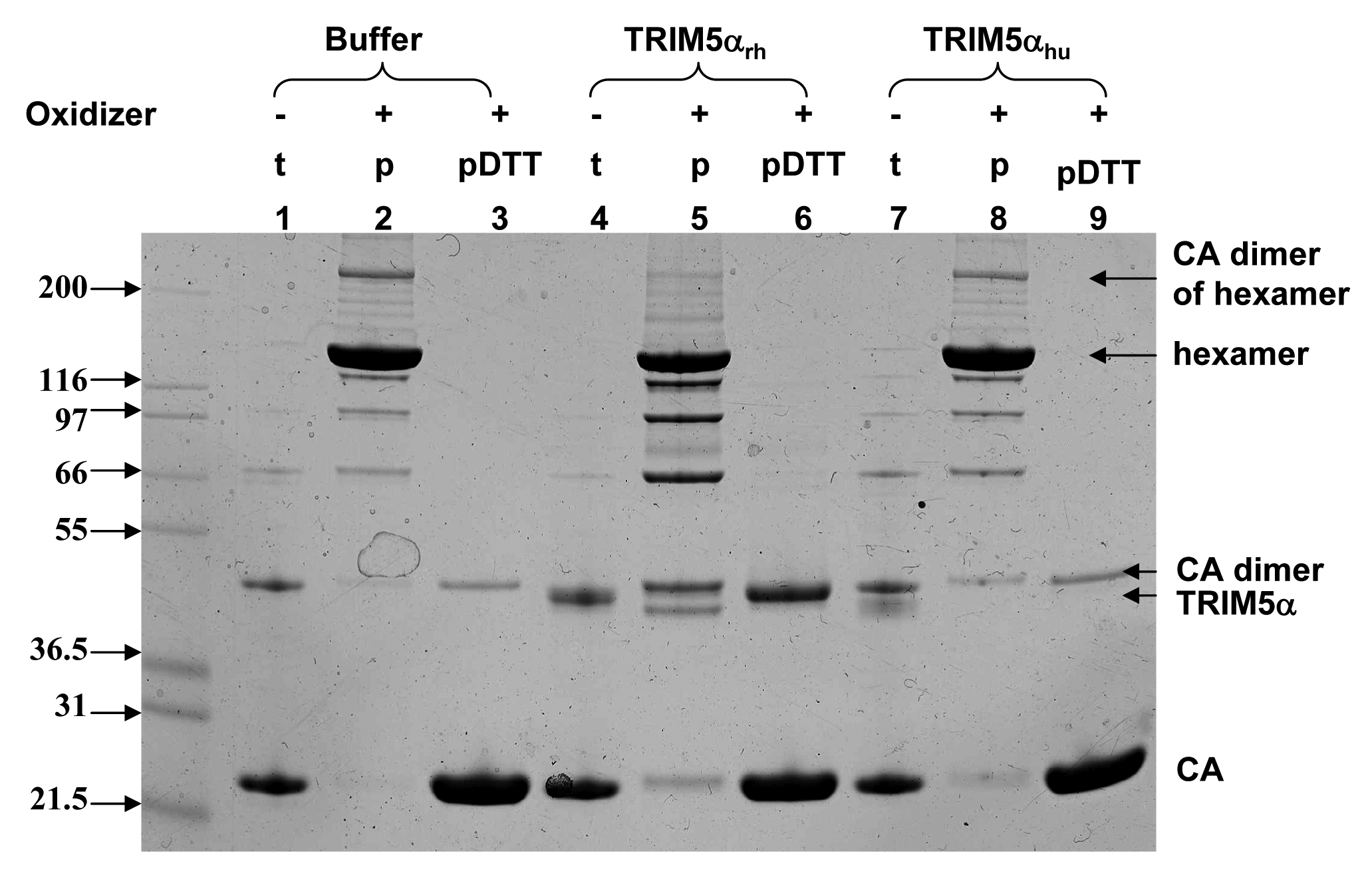

Supplement: Figure S6 — SDS-PAGE analysis of TRIM5αrh CC-SPRY binding to A14C/E45C CA tubes. A14C/E45C CA assemblies were incubated with either TRIM5αhu CC-SPRY, TRIM5αrh CC-SPRY or reaction buffer followed by oxidization. Samples of the reaction mixture before high speed centrifugation (t), and pellets of non-reduced (p) and reduced (pDTT) samples were analyzed by non-reducing SDS-PAGE and stained with Coomassie Blue. A CA dimer is observed (lane 1, 3, 5, 7, 9) in non-oxidized samples, whereas a dimer of CA hexamers is only seen in oxidized A14C/E45C CA assemblies without TRIM5αrh CC-SPRY treatment (lane 2 and 8). (TIF) [file ppat.1002009.s006.tif]

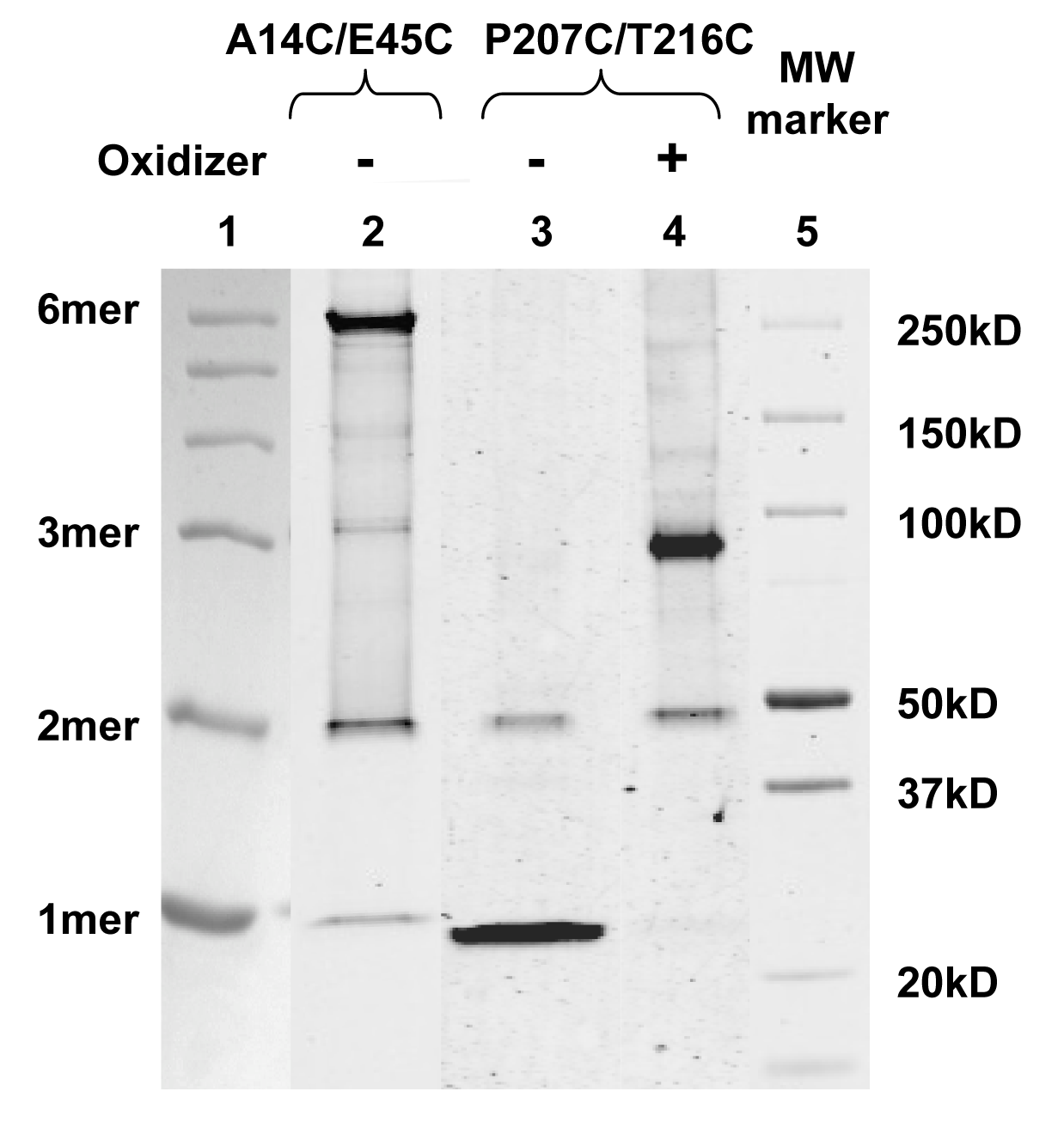

Supplement: Figure S7 — Non-reducing SDS-PAGE analysis of isolated HIV-1 A14C/E45C and P207C/T216C cores, detected by immunoblotting with rabbit anti-CA serum. Lane 1, A CA oligomer ladder formed by purified, cross-linked P17C/T19C CA (gift from Dr. Owen Pornillos [Pornillos O, Ganser-Pornillos BK, Banumathi S, Hua Y, & Yeager M (2010) Disulfide bond stabilization of the hexameric capsomer of human immunodeficiency virus. J Mol Biol 401(5):985-995.]); lane 2, isolated A14C/E45C cores contained predominantly hexameric CA; lane 3, isolated P207/T216C cores contained primarily monomeric CA; lane 4, isolated P207/T216C cores contained primarily CA trimers after oxidization; lane 5, molecular weight markers. (TIF) [file ppat.1002009.s007.tif]
